# Identification of neuronal network properties from the spectral analysis of calcium imaging signals in neuronal cultures

**DOI:** 10.3389/fncir.2013.00199

**Published:** 2013-12-18

**Authors:** Elisenda Tibau, Miguel Valencia, Jordi Soriano

**Affiliations:** ^1^Neurophysics Laboratory, Departament d'Estructura i Constituents de la Matèria, Universitat de BarcelonaBarcelona, Spain; ^2^Neurophysiology Laboratory, Division of Neurosciences, CIMA, Universidad de NavarraPamplona, Spain

**Keywords:** neuronal cultures, multineuron calcium imaging, spectral analysis, network development, excitation-inhibition balance, GABA switch, synchronous oscillations

## Abstract

Neuronal networks *in vitro* are prominent systems to study the development of connections in living neuronal networks and the interplay between connectivity, activity and function. These cultured networks show a rich spontaneous activity that evolves concurrently with the connectivity of the underlying network. In this work we monitor the development of neuronal cultures, and record their activity using calcium fluorescence imaging. We use spectral analysis to characterize global dynamical and structural traits of the neuronal cultures. We first observe that the power spectrum can be used as a signature of the state of the network, for instance when inhibition is active or silent, as well as a measure of the network's connectivity strength. Second, the power spectrum identifies prominent developmental changes in the network such as GABA_A_ switch. And third, the analysis of the spatial distribution of the spectral density, in experiments with a controlled disintegration of the network through CNQX, an AMPA-glutamate receptor antagonist in excitatory neurons, reveals the existence of communities of strongly connected, highly active neurons that display synchronous oscillations. Our work illustrates the interest of spectral analysis for the study of *in vitro* networks, and its potential use as a network-state indicator, for instance to compare healthy and diseased neuronal networks.

## 1. Introduction

Living neuronal networks, from the smallest neuronal assembly up to the human brain, are one of the most fascinating yet intricate structures in Nature. The subtle interplay between the architecture of the neuronal network and the dynamics of the neurons give rise to a vast mosaic of complex phenomena that are still a major paradigm in neuroscience (Bassett and Gazzaniga, 2011), including spontaneous activity patterns (Blankenship and Feller, 2009; Deco et al., 2010; Luczak and MacLean, 2012), information processing and routing (Bullmore and Sporns, 2012), synchronization (Salinas and Sejnowski, 2001), plasticity and adaptability (Destexhe and Marder, 2004), together with remarkable self-organizing properties and critical behavior that suggest an efficient yet flexible *modus operandi* (Chialvo, 2010; Bullmore and Sporns, 2012).

The interplay between single cell dynamics and network topology is tremendously complex, particularly when applied to the comprehension of the human brain (Chicurel, 2000; Alivisatos et al., 2012; Abbott, 2013). However, in the last two decades we have attended to an outbreak in the development of techniques to investigate the brain *in vivo*. Advances in brain functional and mapping techniques such as fMRI, EEG, MEG, or DTI, together with resources from graph theory and signal processing (Bullmore and Sporns, 2009; Feldt et al., 2011), have provided unprecedented detail on brain functional interactions and their dependence with the underlying circuitry. They have also opened new perspectives in our comprehension of dysfunctional circuits. Indeed, severe neurological disorders and behavioral deficits are associated to alterations of the neuronal circuitry (Seeley et al., 2009), abnormal neuronal activity coordination (Uhlhaas and Singer, 2012), or deficient neuronal machinery (Maccioni et al., 2001). Autism, for instance, has been ascribed to an underconnectivity or overconnectivity of local brain circuits combined with long-distance disconnection. Schizophrenia has been associated with an imbalance of the excitatory and inhibitory circuits, among other factors (Lynall et al., 2010; Yizhar et al., 2011b). Epileptic brains, compared to those of healthy subjects, display a richer functional connectivity with a clear modular structure (Chavez et al., 2010), while brain networks in Alzheimer's disease patients are characterized by a loss of the small-world network feature (Stam et al., 2007).

These advances have provided novel clinical prognosis tools by linking specific functional failures to topological traits of the anatomical network. They have evidenced that the information obtained from functional and anatomical techniques contain several signatures that reveal the properties of brain functions, both in normal and disease states. Nevertheless, a major difficulty in analyzing this information has been the sheer size and complexity of the human brain. The activity recorded from the intact brain results from the occurrence of several, simultaneous processes involving a huge number of interacting cells, thus complicating the understanding of the ultimate mechanisms that regulate neural activity. These difficulties have called for more controlled, accessible and simplified systems that would allow to investigate the basis of brain operation. Neuronal cultures have emerged as one of those systems. These *in vitro* preparations are typically derived from dissociated rat cortical or hippocampal tissues, can be maintained for several months, and their activity monitored by a number of recording techniques that are able to track single cell behavior (Eckmann et al., 2007). The flexibility of neuronal cultures to fit diverse experimental platforms, as well as the ability to *act* on them by chemical, electrical or other means, have made them very attractive for a large number of investigations, most notably the emergence and richness of spontaneous activity patterns (Wagenaar et al., 2006a; Orlandi et al., 2013), the interplay activity-connectivity (Volman et al., 2005), the network's self-organizing potential (Pasquale et al., 2008), and criticality (Tetzlaff et al., 2010).

Here we propose to use analytical tools based on spectral analysis to investigate the functional and structural topology of neural cultures. We use fluorescence calcium imaging to monitor the spontaneous activity of the neuronal network with single cell resolution. In a first set of experiments, we investigate the development of the network along the first 3 weeks of maturation, a period in which the average neuronal connectivity, circuitry topology, and the excitatory-inhibitory balance change significantly. In a second set of measurements, we perturb the topology of a mature culture by gradually weakening the excitatory connections. This action results in a gradual decay of collective spontaneous activity until it is fully disrupted. The analysis of the power spectrum in these two scenarios evidences that spectral data can capture dynamical features of the neuronal network. Our study is a preliminary investigation that, although it requires a thorough exploration and modeling, may help understanding the use of statistical descriptors to detect and quantify distinct topological and dynamical traits in neuronal networks.

## 2. Materials and methods

### 2.1. Neuronal cultures

Rat cortical neurons from 18 to 19-day-old Sprague-Dawley embryos were used in the experiments. All procedures were approved by the Ethical Committee for Animal Experimentation of the University of Barcelona, under order DMAH-5461. Following standard procedures described in previous studies (Soriano et al., 2008; Orlandi et al., 2013), dissection was carried out in ice-cold L-15 medium (Life) enriched with 0.6% glucose and 0.5% gentamicin (Sigma-Aldrich). Embryonic cortices were isolated from the rest of the brain and neurons dissociated by pipetting.

Cortical neurons were plated on 13 mm glass coverslips (#1 Marienfeld-Superior). Prior to plating, glasses were washed in 70% nitric acid for 2 h, rinsed with double-distilled water (DDW), sonicated in ethanol and flamed. To facilitate a homogeneous distribution of neurons in the cultures, glasses were coated overnight with 0.01% Poly-l-lysine (PLL, Sigma). Cultures were incubated at 37°C, 95% humidity, and 5% CO_2_ for 4 days in plating medium [90% Eagle's MEM—supplemented with 0.6% glucose, 1% 100X glutamax (Gibco), and 20 μg/ml gentamicin—with 5% heat-inactivated horse serum, 5% heat-inactivated fetal calf serum, and 1 μl/ml B27]. The medium was next switched to changing medium [90% supplemented MEM, 9.5% heat-inactivated horse serum, and 0.5% FUDR (5-fluoro-deoxy-uridine)] for 3 days to limit glia growth, and thereafter to final medium [90% supplemented MEM and 10% heat-inactivated horse serum]. The final medium was refreshed every 3 days by replacing half of the culture well volume. Plating was carried out with a nominal density of 1 million cells/well (5000 neurons/mm^2^), providing a final density in the range 200–400 neurons/mm^2^.

Cultures prepared in these conditions contain both excitatory and inhibitory neurons, whose strength can be controlled by the application of 6-cyano-7-nitroquinoxaline-2,3-dione (CNQX, Sigma), an AMPA-glutamate receptor antagonists in excitatory neurons; or through bicuculine-methbromide (Sigma), a GABA_A_ receptor antagonist in inhibitory neurons.

### 2.2. Preparation of the experiments

Our study encompassed two groups of experiments. In a first one we monitored neuronal activity along the maturation of the network; in a second one we studied the disintegration of the network by gradually blocking AMPA-excitatory connections through CNQX.

The study of the evolution of the network as a function of the culture age (days *in vitro*, DIV) started with the preparation of 2–3 batches that contained 24 identical cultures each. One of the batches was next selected for analysis, which was carefully inspected before the beginning of the series of measurements. We used only those batches whose cultures contained a similar number of neurons, and homogeneously distributed over the substrate. Measurements then consisted in the systematic recording of spontaneous activity in the cultures of the batch, in 24 h intervals along 3 weeks.

We verified that the culture medium changes did not biased the results presented here, particularly those related with the maturation of the network. This verification was carried out by measuring neuronal activity along 2 weeks in batches where we either replaced completely the mediums in each change, or in batches where we replaced only 1/3 of the culture well volume. All development experiments showed the same trend within experimental error, independently of the medium change protocol.

The disintegration experiments were also carried out in cultures that were prepared and inspected as the above. As described later, we considered cultures in the range 8–16 DIV, which were sufficiently mature to show rich spontaneous activity during the different stages of disintegration.

### 2.3. Experimental setup

Measurements consisted in the recording of spontaneous activity through calcium imaging, which allows the monitoring of neuronal firing by the binding of Ca^2+^ ions to a fluorescent indicator (Grienberger and Konnerth, 2012). Prior to imaging, cultures were incubated for 40 min in External Medium (EM, consisting of 128 mM NaCl, 1 mM CaCl_2_, 1 mM MgCl_2_, 45 mM sucrose, 10 mM glucose, and 0.01 M Hepes; treated to pH 7.4) in the presence of the cell-permeant calcium sensitive dye Fluo-4-AM (Gee et al., 2000), with 4 μl Fluo-4 per ml of EM. The culture was washed with fresh EM after incubation and finally placed in a recording chamber containing 4 ml of EM.

The recording chamber was mounted on a Zeiss inverted microscope equipped with a 5X objective and a 0.32X optical zoom. Spontaneous neuronal activity was monitored through a Hamamatsu Orca Flash 2.8 CMOS camera attached to the microscope, in combination with a light source for fluorescence. Images were acquired with a speed of 20 or 33 frames per second (respectively, 50 or 30 ms interval between two consecutive frames) and a spatial resolution of 4.40 μm/pixel. Images had a size of 960 × 720 pixels with 256 gray-scale levels. This settings provided a final field of view of 4.2 × 3.2 mm^2^ that contained on the order of 3000 neurons. Camera, microscope and light source settings were optimized to minimize photo-bleaching and photo-damage while providing the best signal to noise ratio throughout the measurements.

### 2.4. Experimental procedure and pharmacology

For the experiments where we investigated the development of the network, we proceeded as follows. We first recorded spontaneous activity as a long sequence of images with a total duration of 30 min, with both excitation and inhibition active (*“E + I” network*). We next fully blocked inhibitory synapses with 40 μM bicuculline, a GABA_A_ antagonist, so that activity was solely driven by excitatory neurons (*“E-only” network*). We then left the culture in darkness for 10 min for the drug to take effect, and finally measured again for 30 min with identical experimental settings.

For the experiments where we monitored the disintegration of the network, we first completely blocked inhibition with 40 μM bicuculline as well as NMDA receptors with 20 μM APV. We then waited 10 min and measured spontaneous activity for 20 min (“E-only” activity). Next, we started a sequence of gradual application of CNQX, and explored concentrations of 50, 100, 200, 400, 800, and 2000 nM. After each application we waited 5 min for the drug to take effect, and measured spontaneous activity for 15 min. The total duration of the experiment was about 2 h. We verified by washing off the drug and measuring again “E-only” network activity that the culture health was not compromised by the long duration of the experiment. Other studies that used almost identical disintegration protocols confirmed the good health of the culture throughout the experiment (Soriano et al., 2008; Jacobi et al., 2009).

In all experiments we also quantified the background signal of the recording system to assess our ability in resolving neuronal firings from actual noise. To do this, we removed the culture from the recording chamber and measured the noise of the camera as well as possible additional artifacts, such as fluctuations in the light of the fluorescence lamp or contamination from indirect light sources in the laboratory. We finally verified that the results presented here were not influenced by any artifact from the experimental system.

### 2.5. Data analysis

**Figure 1:**
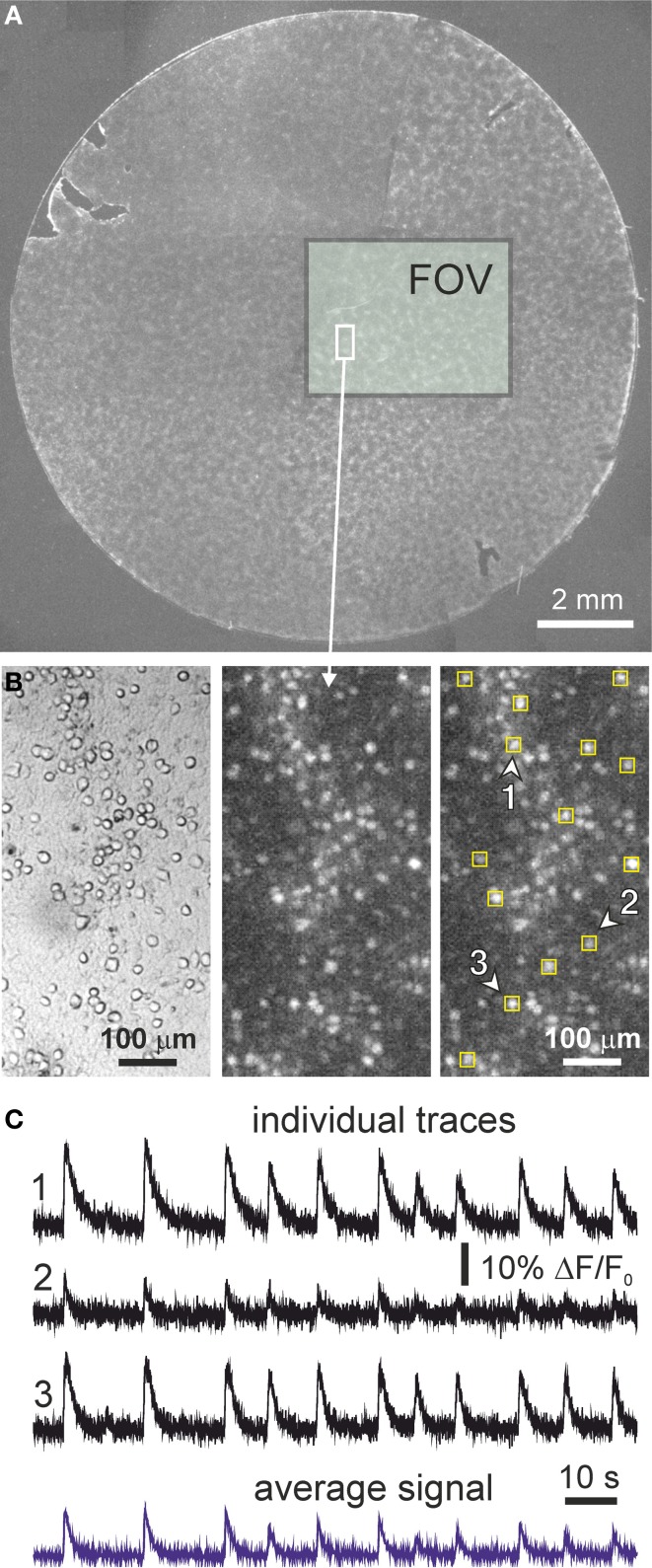
**Cortical cultures and neuronal activity**. **(A)** Example of an entire neuronal culture 13 mm in diameter at day *in vitro* 9. The rectangular box shows the actual field of view (FOV) of the camera and illustrates the size of the monitored area. **(B)** Detail of a small region of the monitored area containing about 100 neurons. Images correspond to bright field (left) and fluorescence (center), together with an example of the selection of the regions of interest for monitoring single neuronal activity (right). The fluorescence image has been integrated over 100 frames, and bright spots correspond to firing neurons. **(C)** Illustrative fluorescence traces of neuronal activity in three regions of interest (labeled and marked with arrowheads in the above images) along 2 min. Fluorescence traces are expressed as Δ *F*/*F*_0_ (background corrected fluorescence divided by the resting fluorescence). Both excitation and inhibition were active during the recording (“E+I” networks). Note the variability in fluorescence amplitude from neuron to neuron. The bottom trace in blue shows the fluorescence signal averaged over all the neurons in the field of view.

At the end of each experiment we took bright-field images for a better identification of the neuronal cell bodies (see Figure 1). We then manually marked each neuron as a squared region of interest (ROI) with a typical lateral size of 10 pixels (about 40 μm). Each experiment typically contained about 2000 ROIs, i.e., individual neurons. The analysis of the average gray level in each ROI along the entire acquired image sequence finally provided the fluorescence intensity *F* for each neuron as a function of time.

Long trains of neuronal activity may contain a small drift of the baseline signal due to photo-bleaching. Although we observed such an effect only in about 5% of the neurons, we automatically corrected this artifact by applying a moving median filter of width 2000 points. We verified that such a correction did not modify the shape of neuronal signal during firing events.

Finally, the fluorescence trace *F(t)* was normalized for each neuron to correct for its background brightness level by computing $\tilde{F}$(*t*) = (*F(t*)−*F*_0_)/*F*_0_ ≡ Δ*F*/*F*_0_, where *F*_0_ is the average amplitude of the background fluorescence signal at rest. The illustrative traces of Figure 1, as well as all the data shown in this work, correspond to such a corrected data.

Neuronal activity in our cultures is characterized by episodes of intense, network-spanning activity events (bursts) combined with quiescent interval of erratic individual firing. The interval between bursting episodes was calculated over the average signal of the neuronal network to take advantage of the almost synchronous bursting episodes. We first determined the onset time of neuronal activation, which was achieved by detecting those events in the fluorescence signal that were at least four times above the standard deviation of the signal. Second, we computed the difference between consecutive onset times, to finally provide the interburst interval distributions.

### 2.6. Spectral analysis

To analyze the spectral content of the fluorescence signals, we computed the power spectral density of the normalized traces $\tilde{F}$(*t*) = Δ*F*/*F*_0_ by using the Welch periodogram method (Welch, 1967; Halliday et al., 1995) implemented in Matlab 7.12.0. Signal is divided into Hamming windows of 256 points (approximately 10 s), 50% overlapped. To estimate the FFT, 1024 points are used, applying zero-padding. Because we use a sample frequency of 20 Hz for young cultures and 33 Hz for mature culture, the frequency resolution is of 0.019 Hz and 0.032 Hz, respectively. The corresponding frequency ranges are (0.078–10) Hz and (0.128–16.5) Hz. Finally, the averaged spectrum for the whole set of neurons was computed when required, for instance to compare global network characteristics during the maturation of the cultures.

For the studies where we investigated the spatial distribution of the *local* energy across the different frequencies we calculated—for each neuron—the average signal of the selected neuron and its *n* = 100 closest neighbors. Then, the resulting time-series were analyzed following the same procedure described above. By plotting the spectral energy of each neuron at a frequency of interest we obtained a two-dimensional representation of spectral energy that revealed those neurons or groups of neurons with the strongest power at that frequency of interest.

The smoothing of the fluorescence signal by averaging with neighboring cells significantly reduced the noise of the PSD data. We tested different *n* values and observed that 100 was the appropriate value to balance a neat PSD signal and low overlap, particularly in the studies of spatial distribution of spectral energy. For the latter, we indeed verified that the results did not change significantly up to *n* ≃ 500.

## 3. Results

### 3.1. Neuronal cultures and network activity

The neuronal networks that we study are constituted by an ensemble of thousands of neurons that have been dissociated from rat cortical tissue and homogeneously plated on glass cover slips 13 mm in diameter, as shown in Figure 1A and described in detailed in the Materials and Methods section. Neurons grown in these conditions have a remarkable self-organizing potential, connecting to one another within hours and showing spontaneous activity as early as day *in vitro* (DIV) 4–6 (Chiappalone et al., 2006; Pasquale et al., 2008; Soriano et al., 2008). Although neurons develop in a relatively large area, with our imaging instrumentation we observe a small but representative region of 13.4 mm^2^ that contains few thousand neurons. A detailed inspection of our cultures reveal their spatial distribution which, despite some clustering, is compatible with a homogeneous distribution of neurons (Figure 1B). We monitor neuronal activity with fluorescence calcium imaging. As shown in the panels of Figure 1B, the spatial resolution of our measuring device is sufficient to trace the behavior of all the neurons in the field of view, with single-cell resolution, and along several hours.

Figure 1C provides examples of fluorescence traces in our cultures, for measurements with both excitation and inhibition active (“E+I” networks). The traces correspond to a developing culture at DIV 9. Fluorescence displays a fast onset due to neuronal activation, followed by a slow decay back to the baseline and that corresponds to the slow unbinding rate of calcium ions from the fluorescent probe.

Neuronal network activity in cultures is characterized by episodes of collective neuronal activation termed *bursts* where the neurons fire in a quasi-synchronous manner in a short time window of ~200 ms. Almost the entire population of neurons participate in a bursting episode, which is observed in the traces of Figure 1C by the quasi-simultaneous occurrence of firing across the neurons. The timing of the bursts themselves is in general regular, with average interburst intervals on the order of 10 s in the provided example. In between bursts, neuronal activity is characterized by sparse, asynchronous firings across the network.

The properties of spontaneous activity, and in particular the structure of the bursting episodes, depends both on the excitability of the neurons, i.e., their ability to spontaneously fire, and the connectivity of the network, i.e., the ability to recruit, amplify and propagate activity from other neurons. The latter is particularly important since connectivity significantly changes during the maturation of the network.

### 3.2. Network development

To investigate distinct features of spontaneous activity due to varying neuronal connectivity, we first treat the scenario in which the network grows and matures along several days *in vitro* (DIV).

**Figure 2:**
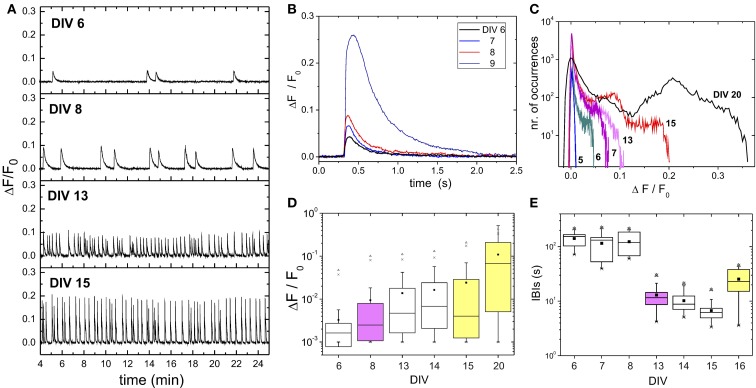
**Network development**. **(A)** Examples of fluorescence traces of spontaneous activity along 15 days of development. All measurements correspond to cultures from the same batch. Traces are the average over the monitored network population (≃ 2000 neurons). The peaks of fluorescence signal identify bursting episodes. The time elapsed between two consecutive bursts define the interburst interval (IBI). The measurement at day *in vitro* (DIV) 6 corresponds to the first observation of spontaneous bursting activity in the batch. **(B)** Detail of a bursting event (averaged over the monitored network population) during the early stages of development to illustrate the substantial increase in fluorescence amplitude after DIV 8. **(C)** Histogram of the network-averaged fluorescence signal for representative stages of development. Bursting activity is absent at DIV 5, giving rise to a fluorescence histogram that is close to a Gaussian distribution. The distributions broaden as bursts emerge and increase in amplitude. **(D)** Box plots of the statistical analysis of the fluorescence distributions. Note the logarithmic scale in the vertical axis. The mean of the distribution (■) and its maximum value (▲) substantially increase by DIV ≳ 8 (pink) and after DIV 15 (yellow), suggesting major evolutionary switches of the network. In the figure, whiskers represent 25 and 75% confidence intervals, and crosses (x) 1 and 99%, respectively. **(E)** IBIs box plot analyses. The broad IBI distribution observed for young cultures significantly changes to a narrow distribution with stable IBI timing after DIV 8, to change again toward a higher variability by DIV ≳ 15.

Neurons in our preparations are plated homogeneously on the glass substrate and lack any initial connectivity. However, development occurred rapidly. We already observed connections as early as 24 h after plating and, consistently with other studies (Soriano et al., 2008), neurons were electrically excitable by DIV 2–3 (data not shown). Spontaneous activity appeared by DIV 5–6, subsequently changing in strength and structure as the culture matured and evolved further. Figure 2 illustrates this behavior for a given culture batch, and with both excitation and inhibition active (“E+I” network). Representative fluorescence traces of average network activity in a period of 15 days of development are provided in Figure 2A. For this batch we observed the first occurrence of bursting at DIV 6. At earlier days, the bursting dynamics was either absent or too sparse to be detected. Although the presence of bursts is clear at DIV 6, their interburst timing is irregular and the firing amplitudes low. By DIV 8 the fluorescence amplitude has substantially increased and bursting has become more regular, reaching a stage of high periodicity by 2 weeks after plating. At later stages of development we observed different trends from batch to batch, with firing amplitudes and interburst intervals stabilizing or decreasing.

Figure 2B depicts the shape and strength of a burst among different evolutionary stages. Bursts are time-shifted for the onset of network activation to coincide. The plot reveals the gradual increase in bursting amplitude during the early stages of development, and the sudden jump at DIV 9, which hints at strong changes in both neuronal excitability and network connectivity.

The example of Figure 2B highlights the dominance of the burst shape (amplitude and width) on the structure of the recorded signal. This is further evidenced in Figure 2C, which shows the distribution of fluorescence amplitudes for the population-averaged signal along maturation. The distribution at DIV 5 is close to a Gaussian distribution, indicating the absence of firing events sufficiently strong to be detected by the camera. As development continued, the histogram of amplitudes became distinctly right-skewed, with progressively higher values of fluorescence. A detailed statistical analysis of the changes in fluorescence is provided in Figure 2D, and illustrates the strong asymmetry of the fluorescence distributions. Interestingly, the major changes in firing amplitude occur by the end of the first and second weeks *in vitro*. The average firing amplitudes (denoted by a black square) as well as the maximum measured amplitudes (up triangles) abruptly jump at these stages.

These changes in network dynamic behavior are also captured by the distribution of interburst intervals (IBIs), which show a tendency to become well timed as the cultures mature (Figure 2D). The average IBI reduces from high, broadly distributed values in the range 100–200 s at DIV 5–8 to narrowly distributed values around 10–20 s after DIV 8. By DIV 16 the network dynamics changes again toward a more erratic behavior and larger IBIs.

### 3.3. Emergence of inhibition during development

**Figure 3:**
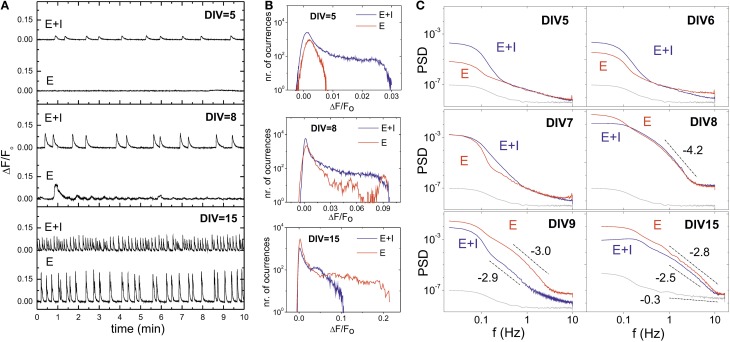
**Influence of inhibitory action during development and GABA switch**. **(A)** Illustrative population-averaged traces of spontaneous activity during development, and comparing “E+I” (top traces) and “E-only” signals (bottom ones) on the same culture. GABA has an excitatory role at early developmental stages and therefore its blockade effectively reduces excitation and silences the network. GABA switches to its normal inhibitory role by DIV ≃ 6 − 7. In maturer cultures, the blockade of inhibition increases excitation and the strength of the bursting episodes. **(B)** Corresponding fluorescence amplitude distributions, depicting the gradual increase in values as maturation progresses. At DIV 8 the blockade of inhibition neither silences the network nor strengthens firing, signaling the GABA switch event. **(C)** Power spectrum densities (PSD) of the spontaneous activity signals, averaged over the monitored population, and along representative stages of development. The gray curve shows the PSD associated to the noise of the camera. The PSD for “E+I” (blue) and “E-only” recordings (red) are markedly different except during GABA switch, at DIV ≃ 7 − 8, signaling its occurrence. The lines and their slopes are a guide to illustrate the markedly different behavior of the PSD between noise and actual measurements.

The role of inhibition during development is depicted in Figure 3. A first interesting feature is the observation that the blockade of inhibition (“E-only” recordings, see Materials and Methods) at early stages of development silences the network or strongly disrupts its activity, as shown in the network-averaged traces at DIV 5 and 8 in Figure 3A. Such a disruption is a consequence of the depolarizing action of GABA at early developmental stages and that confers it an excitatory role (Ben-Ari, 2002). Therefore, the blockade of GABA_A_ effectively reduces excitation and, in turn, the mechanisms for the network to spontaneously fire.

GABA changes to its normal inhibitory action by DIV 7, an event known as GABA switch (Ganguly et al., 2001; Soriano et al., 2008). The blockade of inhibition at this and subsequent stages results in strong bursting due to the excess in excitation, which is revealed by the high fluorescence amplitudes at DIV 15 (Figure 3A).

The distribution of fluorescence amplitudes of Figure 3B also illustrates the changing role of inhibition during development. “E+I” networks show bursting activity already at DIV 5, with broad fluorescence distributions that gradually increase in width as bursts strengthen in maturer stages. “E-only” networks, however, show at DIV 5 a distribution of fluorescences close to a Gaussian distribution, although the slight deviation at high fluorescences hint at some sporadic, individual neuronal activity. Bursting is observed by DIV 7–8, though very erratic due to GABA switch. At the other extreme of development (DIV 15) network behavior completely changes, and the bursting amplitudes in the “E-only” condition are much higher than in the “E+I” one.

In general, the blockade of inhibition in cultures older than 1 week leads to a substantial increase of the fluorescence amplitudes, larger interburst intervals and a higher regularity of bursting episodes. These distinct traits of “E-only” networks are a consequence of the absent firing-regulatory role of inhibition, which causes the neurons to fire until the excitatory neurotransmitter's pool is exhausted (Cohen and Segal, 2011).

We observed that GABA switch could be well identified by analyzing the network average fluorescence signal in terms of the power spectrum density (PSD), and comparing the two network conditions along development. As shown in Figure 3C, at DIV 5 and 6 the “E-only” signal is below the “E+I” one. The spectra for the “E-only” case also scales with lower slopes, indicating a much different behavior of the network, which is either silent or very weak in activity. By DIV 7–8 the spectral curves cross one another. Most likely inhibition has here a mixed role across the culture during the GABA switch event, leading to a similar spectral trend in the two network conditions. GABA is completely inhibitory at DIV 9 and maturer cultures, and the “E-only” curves are now the ones with the highest energy compared to the “E+I” case.

**Figure 4:**
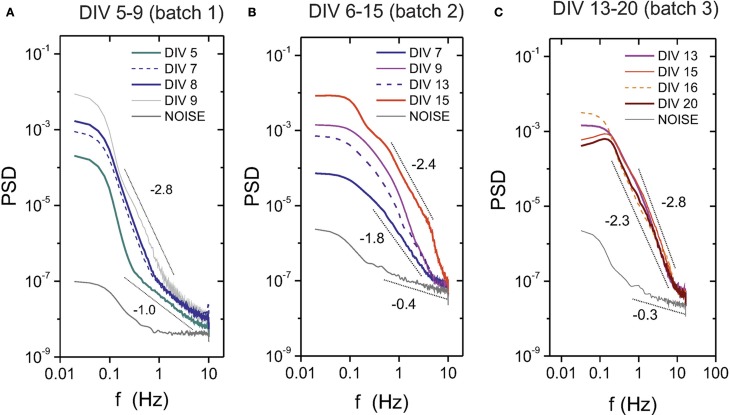
**Power spectrum density (PSD) during development for “E+I” networks**. The plots show the PSD evolution for three different culture batches **(A–C)**, covering in total about 3 weeks of development. The PSD gradually shifts upwards as the network matures, a feature that is accompanied by a tendency of the PSD to scale as a power law *P*~ f^-α^. Mature cultures at DIV ≳ 13 appear close to one another, suggesting that approximately after 2 weeks in *in vitro* cultures have reached a stable stage. The dotted black lines are a guide to the eye to illustrate the increasing values of α along maturation.

We also show in Figure 4 the evolution of the PSD for three different batches and covering different ranges of the maturation process. We show only the “E+I” data to emphasize developmental traits. The plots depict the general trend that the power spectra moves upwards and with progressively higher slopes as the cultures mature and the bursts strengthen. At DIV 5, which corresponds to the first occurrence of bursting activity for this batch, the corresponding PSD curve is distinctly above the noise level. The shape of the PSD curves and their relative shift substantially change during evolution, signaling the progressive increase in bursting amplitudes and frequency. After the second week *in vitro*, however, the cultures seem to reach a stable phase, with all spectra showing similar amplitudes and effectively collapsing into one another. The PSD here fits well a power law behavior *P* ~ *f*^−α^, with 2.3 ≲ α ≲ 2.8.

### 3.4. Network disintegration

**Figure 5:**
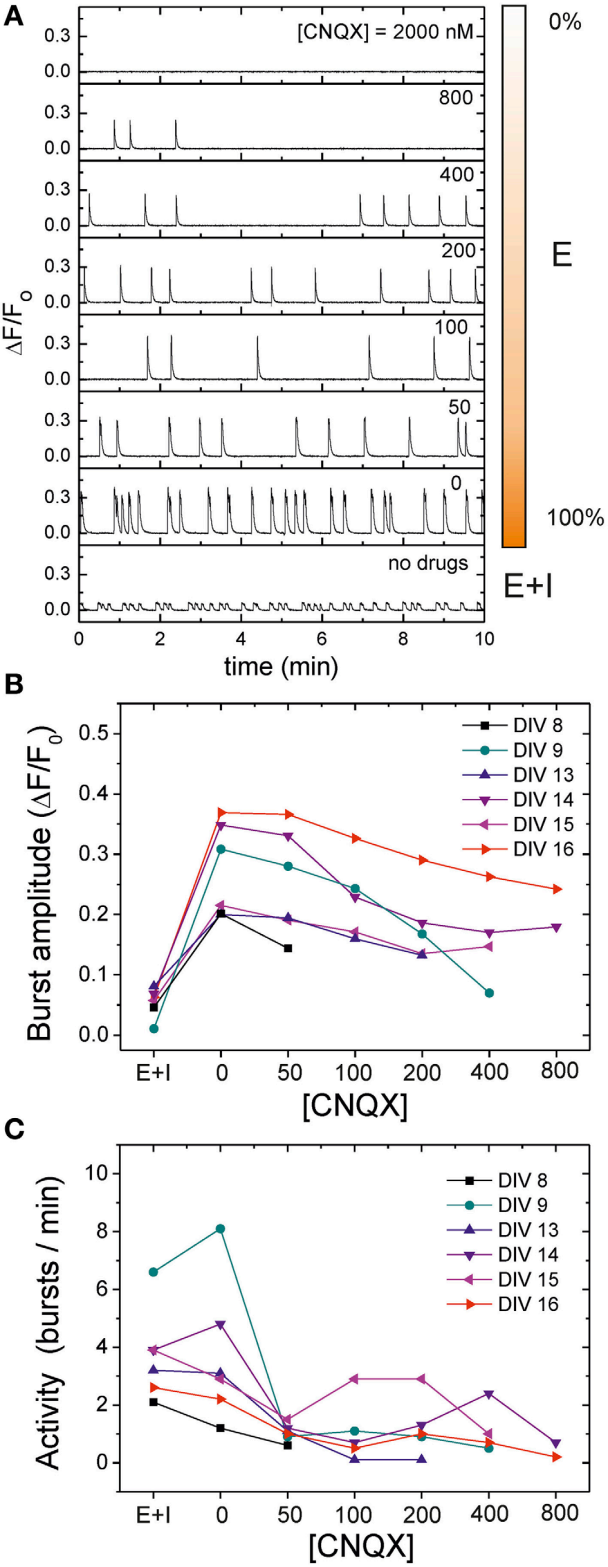
**Network disintegration through CNQX in an “E-only” network**. **(A)** Examples of spontaneous activity traces for 7 concentrations of the AMPA-excitatory antagonist CNQX, in a mature culture at DIV 16. During the disintegration protocol inhibitory synapses are silenced through application of 40 μM bicuculine. NMDA synapses are also blocked by 20 μM APV. Fluorescence traces are compared with the unperturbed, “E+I” case. For [CNQX] = 0, activity is characterized by high amplitudes and large interbust intervals (IBIs) compared to the “E+I” case. As CNQX is applied, both the bursting amplitude and timing change, until all bursting activity disappears for large [CNQX]. The bar at the right side of the traces illustrates the relative strength of the excitatory connectivity. **(B)** Population-averaged bursting amplitudes during the disintegration process and for different culture ages, showing the steady decay in bursting amplitudes as CNQX increases. **(C)** Average network activity, quantified as 1/ 〈IBI〉, during disintegration. Young cultures decrease in activity and become silent at lower CNQX concentrations than maturer cultures. Maturer cultures show a mixed behavior in which activity initially decreases, to abruptly increase for some CNQX concentrations. The data for DIV 16 shown in panels **(B)** and **(C)** correspond to the exemplary traces of panel **(A)**.

Here we investigate the deterioration in spontaneous activity when the excitatory connectivity of the network is progressively weakened by CNQX, an AMPA-glutamate receptor antagonist in excitatory neurons (see Materials and Methods). In these experiments we fully blocked NMDA and GABA_A_ receptors to restrict ourselves to the simplest scenario. Figure 5A illustrates, for a mature culture at DIV 16, the evolution of the average “E-only” spontaneous activity for increasing concentrations of CNQX. We also provide the activity data for the unperturbed, “E+I” network for comparison. For [CNQX] = 0 (full connectivity strength), the network spontaneous activity shows the usual high-amplitude bursting behavior together with the large interburst intervals characteristic of the dynamics solely driven by excitation. Small additions of CNQX mainly disrupt the average interburst interval, which increases remarkably compared to the initial case. As the disintegration progresses, concentrations of [CNQX] ≳ 200 nM modify both the fluorescence amplitude and the interburst intervals. At extreme values of weakening, [CNQX] ≳ 2000 nM, global network activity is very rare or has stopped completely.

While high concentrations of CNQX completely disrupted bursting, i.e., population-spanning coherent activity, we should note that uncorrelated, neuron-to-neuron activity was still present. Although these events were scarce, we systematically detected their presence in the studied cultures.

To investigate variability in culture age, we carried out the same disintegration protocol for cultures at different stages of maturation. As depicted in Figure 5B, the bursting amplitudes in all these cases show a similar trend. Initially, the blockade of inhibition in the transition from “E+I” to “E-only” connectivity ramps up the bursting amplitude to a maximum, but the subsequent gradual network disintegration leads to a progressive decay in amplitudes until bursts disappear altogether.

This general trend in the decay of bursting amplitudes does not hold for the bursting activity of the network, which is quantified as 1/〈 IBI 〉. As shown in Figure 5C, although most of the cultures at DIV ≲ 13 exhibit a gradual decay in activity upon CNQX application, those cultures at DIV ≳ 14 display an increase in activity at specific concentrations of CNQX. This erratic behavior seems indeed a distinct feature of mature cultures, and hints at the existence of network mechanisms in these cultures that promote activity, possibly to compensate the weakening in connectivity. Moreover, the fact that the increase in activity upon CNQX application occurs at different concentrations from one culture to another may indicate that development drives each culture to slightly different circuit architectures and connectivity strengths.

**Figure 6:**
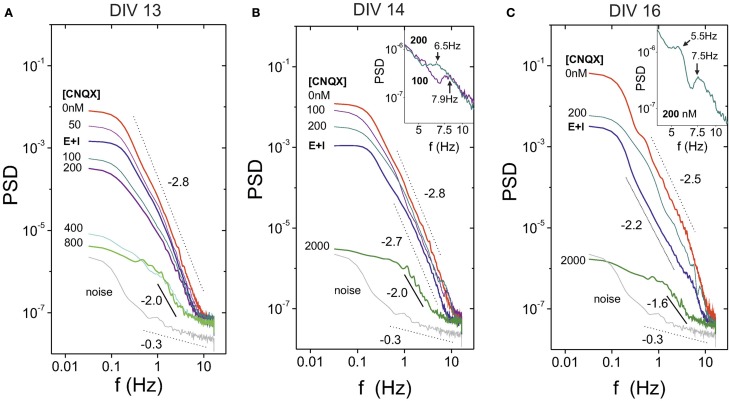
**Power spectrum density (PSD) in mature cultures during network disintegration through CNQX**. **(A)** General trend of the PSD for a culture at DIV 13. For [CNQX] = 0 the PSD exhibits its highest energy, which is associated to the high amplitude of the bursting episodes. The PSD energy gradually decreases with CNQX, and for [CNQX] ≳ 800 nM bursting is absent and the PSD reveals temporal correlations arising from individual neuronal activity. PSD structure at this extreme concentrations is clearly different from the background noise. **(B)** A culture at DIV 14 showing peaks in the PSD at 7 and 8 Hz, which correspond, respectively, to [CNQX] = 200 and 400 nM. The inset provides a detail of the peaks. **(C)** A culture at DIV 16 showing the presence of two peaks, at 5.5 and 7.5 Hz, for [CNQX] = 200 nM. The inset shows a detail of the peaks.

The study of the disintegration process in terms of the PSD is shown in Figure 6A for a culture at DIV 13. This figure portrays the general trend observed in most of the experiments. The PSD initially increases from the “E+I” condition to the “E-only” one due to the large amplitude of the bursts in the absence of inhibition. Next, the gradual addition of CNQX decreases the overall power as well as the PSD slope, concurrently with the progressive decay in bursts amplitudes. However, for large concentrations of CNQX—and rare or inexistent bursting—the PSD exhibits a scaling trend that is distinctly different from both the bursting behavior and the background noise. This scaling suggests that the PSD is capturing temporal correlations between neurons' individual firing events. We note that these neuron-to-neuron interactions could not be detected in measurements with strongest connectivity strengths ([CNQX] ≲ 400 nM) due to the dominance of bursting behavior in network activity.

This general trend actually showed some interesting variations, illustrated in Figures 6B,C. For the example at DIV 14 (Figure 6B) we observed evidences of peaks in the PSD at frequencies *f* ≃ 7−8 Hz. These peaks were particularly strong at CNQX concentrations of 100 and 200 nM. Remarkably, these concentrations also correspond to the ones in which network activity increases upon disintegration. Indeed, we systematically observed a correlation between those experiments in which activity increased at specific values of CNQX and the presence of peaks in the PSD. Another example is provided for a culture at DIV 16 (Figure 6C). In this case we observed two peaks (at around 5 and 7 Hz) for [CNQX] = 200 nM, the concentration at which network activity increases for this culture.

### 3.5. Network spatial traits

**Figure 7:**
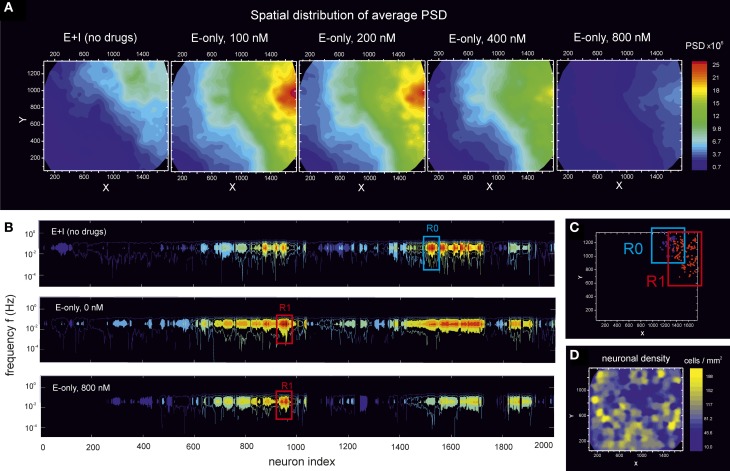
**Distribution of spectral energy across a culture during disintegration through CNQX**. Data corresponds to the experiment at DIV 16 shown in Figure 6C. **(A)** For a given neuron, its PSD (averaged over frequencies) is displayed in the (*x, y*) space according to the spatial location of the neuron. The analysis is then extended to cover all the ≃ 2000 monitored neurons in the field of view, finally providing a color map of the PSD distribution across the network. For the unperturbed, “E+I” network, the neurons with the highest PSD form a compact spot in the top-right corner of the field of view. The blockade of inhibition both shifts and increases the size of this “highly energetic” group of neurons. This region progressively reduces in size as CNQX is applied, but maintains a spatial cohesion up to the complete disintegration of the network. **(B)** Color coded PSD values as a function of the frequency *f* and the neuron index. Neurons are ordered by spatial proximity to highlight two groups of “highly energetic” neurons, termed R0 and R1, that maintain their strong energy and spatial cohesion during disintegration. **(C)** Spatial location of the R0 and R1 neuronal ensembles in the field of view. **(D)** Neuronal density map, calculated by counting the number of neurons in a square unit area 100 μm wide. The high energetic groups R0 and R1 do not correlate with an area of the network particularly populated.

To further explore the PSD potential in characterizing neuronal network features, we analyzed the spatial distribution of spectral energy across the culture. We first considered the average energy, i.e., the mean value of the PSD distribution. Figure 7A shows the map of spectral energy for the PSD data of the culture at DIV 16 depicted in Figure 6C. Spectral energy is shown for the “E-only” condition along different stages of disintegration. The “E+I” data is also provided for reference.

We note that, by considering the entire spectral energy, the PSD values are dominated by the low frequency contributions, i.e., those associated with the amplitude of the bursts. Hence, the map of spectral energy in these conditions effectively shows the distribution of bursting amplitudes across the network.

An interesting feature of the map shown in Figure 7A is that the distribution of energy is inhomogeneous. Neurons with high bursting amplitudes are concentrated in the top-right corner of the field of view, and constitute by themselves a group of spatially close neurons that fire together with similar amplitudes, a quality that is maintained even at high levels of disintegration. We also note that in the transition from “E+I” to “E-only” connectivity, the spatial location of the “highly energetic” neurons substantially changes, evidencing that the balance between excitation and inhibition plays an important role in shaping network's local dynamical features.

The physical closeness of these “highly energetic” neurons is emphasized in Figure 7B, which shows the spectral energy as a function of the neuron index, with neurons ordered by spatial proximity. The plot marks two particularly relevant communities, labeled R0 and R1, whose containing neurons maintain a high spectral energy up to complete disintegration of the network. The location of these two groups in the monitored region of the culture is shown in Figure 7C. We remark that we monitor only a small region of the culture. Therefore, these groups of neurons may also share some traits with (or their dynamics influenced by) other neurons outside the field of view. For sake of discussion, we also provide in Figure 7D the neuronal density map, which highlights those regions in the field of view that are more densely populated. A direct comparison with Figure 7C shows that the two communities R0 and R1 of energetic neurons do not correlate with particularly dense areas, revealing the importance of non-local phenomena (both in circuitry and dynamics) in shaping specific neuronal activity traits.

We carried out this spatial analysis with all the monitored cultures, and covering from very young (DIV 5–6) to mature (DIV ≃ 20) cultures. In general we observed that young cultures up to DIV ≃ 10 displayed a rather homogeneous spatial disintegration, with no identifiable “highly energetic” communities. However, for cultures at DIV 14 and older we systematically observed an inhomogeneous disintegration combined with the existence of communities. The location of these communities varied from culture to culture, and confirmed that mature cultures break the initial network isotropy and develop slightly different connectivity layouts.

### 3.6. Coherent neuronal oscillations

Figures 6B,C introduced the observation that some cultures had a PSD characterized by the presence of peaks at frequencies *f* in the range 5–10 Hz. These peaks were stronger at specific concentrations of CNQX, suggesting the emergence—or reinforcement—of collective oscillatory modes in the network for a precise coupling strength between neurons.

**Figure 8:**
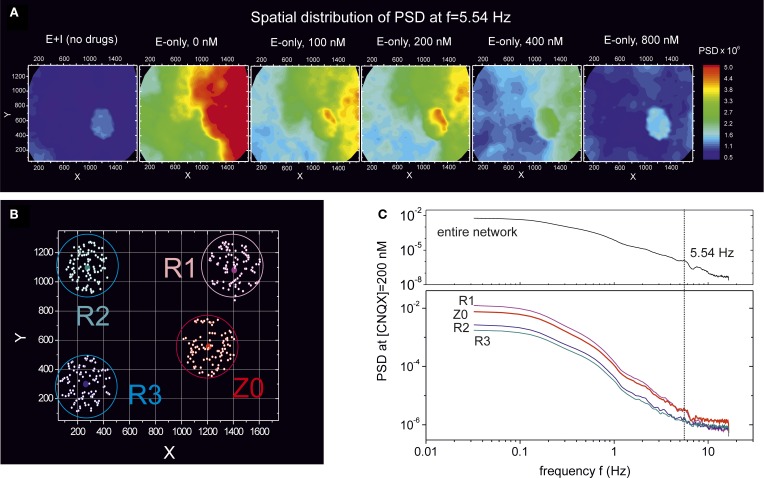
**Emergence of synchronous oscillations during network disintegration through CNQX**. Data corresponds to the experiment at DIV 16 shown in Figure 6C. **(A)** Spatial distribution of the PSD at a frequency of 5.54 Hz and for different connectivity conditions, “E+I” and “E-only” with gradual weakening. The presence of a compact spot at the center-right of the PSD map highlights a neuronal community (termed Z0) that synchronously oscillates at this frequency. Oscillations with strong amplitude also appear along the right edge. For [CNQX] = 200 nM the Z0 community displays the highest difference in energy compared to the neighboring regions. **(B)** Location of 4 different communities. For each community, the central dot marks the position of a selected neuron whose power spectrum is averaged over all the 100 closest neurons (white dots within a circle). **(C)** PSD of the four communities for the “E-only” connectivity at 200 nM. The community Z0 and R1 show a clear peak in the PSD at *f* = 5.54 Hz. The PSD at this frequency is also higher in these two communities compared to the others.

To further investigate these oscillatory modes, we considered again the experiment at DIV 16 whose PSD is shown in Figures 6C, 7. Here, however, we analyze the PSD properties at the frequency *f* = 5.54 Hz, where a peak was well identifiable at [CNQX] = 200 nM. Figure 8A shows the spatial distribution of energy at this frequency for the two network conditions, “E+I” and “E-only”, as well as along gradual disintegration through CNQX.

We first note the remarkable contrast in the spatial distribution of energy at *f* = 5.54 Hz between the “E+I” and “E-only” conditions. The former shows a compact spot of energetically similar neurons, while the latter displays an almost symmetric coverage, with a low energy region on the left that contrasts with a high energy one on the right. Again, these distinct maps reveal the importance of inhibition in shaping network dynamics.

Second, the study also reveals the evolution of this highly energetic spot throughout weakening. Indeed, for the “E+I” condition, the difference in energies between this spot and the neighboring areas is relatively small, by 10%, which made difficult its detection in the PSD of Figure 6C. As the connectivity of the network shifts to the “E-only” condition and CNQX is applied, we observe that the difference between the energy in this spot and its neighborhood ramps to about 45% at [CNQX] = 200 nM, a difference that progressively decreases as the disintegration progresses, although the compactness of the spot is well maintained.

We additionally investigated in more detail the differences in the PSD between the observed compact spot and the neighboring areas. For simplicity, we restricted the analysis to the “E-only” connectivity condition at [CNQX] = 200 nM weakening. Figure 8B depicts four investigated communities. In each community we selected a central neuron and averaged its PSD with the 100 closest neighbors (white dots within a circle in Figure 8B). We label as Z0 the community that corresponds to the “spot” mentioned above, and by R1–R3 the rest of communities. The corresponding PSD distributions are shown in Figure 8C together with the average over the entire network for clarity. We first note that the Z0 and R1 communities have a much higher energy than the others, and that both are markedly characterized by a peak in the PSD at 5.54 Hz. This peak is difficult to observe in the other communities. By comparing these results with the network-averaged PSD, we conclude that both Z0 and R1 are the main contributors to the observed peak at 5.54 Hz, and that Z0 is the community that remains highly coupled throughout disintegration.

**Figure 9:**
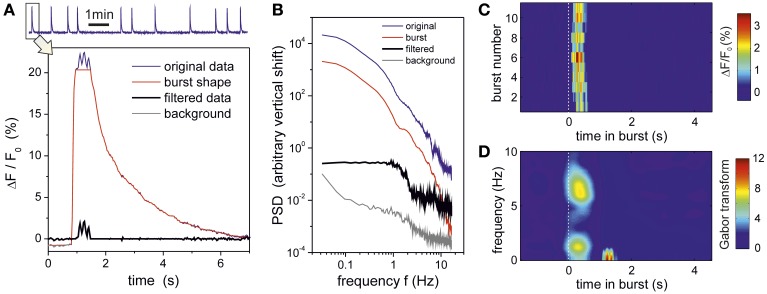
**Oscillations originate in the bursts**. Data corresponds to the experiment at DIV 16 for [CNQX] = 200 nM. **(A)** The top trace depicts the average fluorescence time series of a group of 100 bursting neurons that constitute the *Z*0 community. The first burst of this series and its manipulation is shown in detail in the bottom panel. The blue trace corresponds to the original fluorescence signal, and reveals a well pronounced oscillatory behavior at the peak of bursting. The original signal is separated into two contributions: the burst shape (red) and the oscillatory signal (black). Burst shape is estimated by applying a median filter with length *L* = 33 frames (1 sec); the resulting trace follows the slow dynamics of the burst while the oscillatory signal keeps the higher frequency components. Activity out of the busting episodes (background signal, gray trace) is computed by connecting the periods in between bursting events. **(B)** The spectral analysis of the resulting signals reveals that the burst trace dominates the shape of the power spectrum, and actually keeps the greatest fraction of energy from the original signal. The oscillatory component has a much lower energy but retains network activity correlations (in the range 1 − 5 Hz approximately) as well as the oscillations at 5.5 and 7.5 Hz. The signal out from the bursting episodes does not exhibit any oscillatory components. Curves are vertically shifted a factor 50 from one another (and using the background signal as reference) to better highlight the different shapes of the power spectrum. **(C)** Fluorescence trace of the oscillatory signal for all the 11 bursting episodes of the recording, locked to the initiation of each episode (dashed white line), showing that oscillations originate within the bursts themselves. **(D)** Their corresponding averaged Gabor transform, picturing the presence of an oscillation in the 5–7 Hz range that only appears once the bursts have reached their maximum amplitude and start the decaying phase.

To gain insight into the origin of these synchronous oscillations, we also carried out an analysis in which we investigated the link between the oscillations and the bursts themselves. As shown in Figure 9A, we first separated the original fluorescence signal into two contributions, one containing the low–frequency modulation associated to the shape of the bursts, and another one containing the rest of the signal. The corresponding PSD analysis (Figure 9B) revealed that the shape of the bursts dominates the behavior of the spectral curves and therefore masks the dynamics of the network. On the contrary, the PSD of the filtered data retains both the dynamical traits of the network and completely captures the oscillatory behavior. We also investigated the properties of the signal in between bursting episodes, and excluded any contribution of the background signal to the presence of the oscillations. We therefore confirmed that the oscillations occurred concurrently with the bursts themselves. This is highlighted in Figure 9C, which compares the traces of the filtered signal along the different bursts. In all cases, the onset of the oscillatory behavior practically coincides with the beginning of bursting (*t* = 0s in the plots). The frequency analysis of these traces (averaged over all the bursting episodes) is shown in Figure 9D, revealing a peak at 5–7 Hz, i.e., the range of the initially described characteristic frequencies.

We extended all the above analyses to other cultures characterized by peaks in the PSD. We observed qualitatively similar traits, i.e., the existence of communities with markedly strong synchronous oscillations, the presence of specific CNQX concentrations at which the strength of the oscillatory mode was maximum, and the link between oscillations and bursts. The frequencies of the oscillatory modes as well as their spatial distribution significantly varied among cultures and developmental ages, emphasizing again the formation of specific network features during maturation.

### 3.7. Unhealthy cultures

Figure 6 showed that the PSD could capture, in a regime of suppressed bursting, temporal correlations between individual neuronal firings. Such a burst elimination was achieved by significantly reducing neuronal coupling through CNQX. Based on this observation, we hypothesized that such a network-spanning affectation could also occur in conditions where the health of the culture was compromised. To test such a possibility, we carried out a simple test in which we left the cultures to degrade, at the end of a normal experiment, by leaving them in the recording system for several hours.

**Figure 10:**
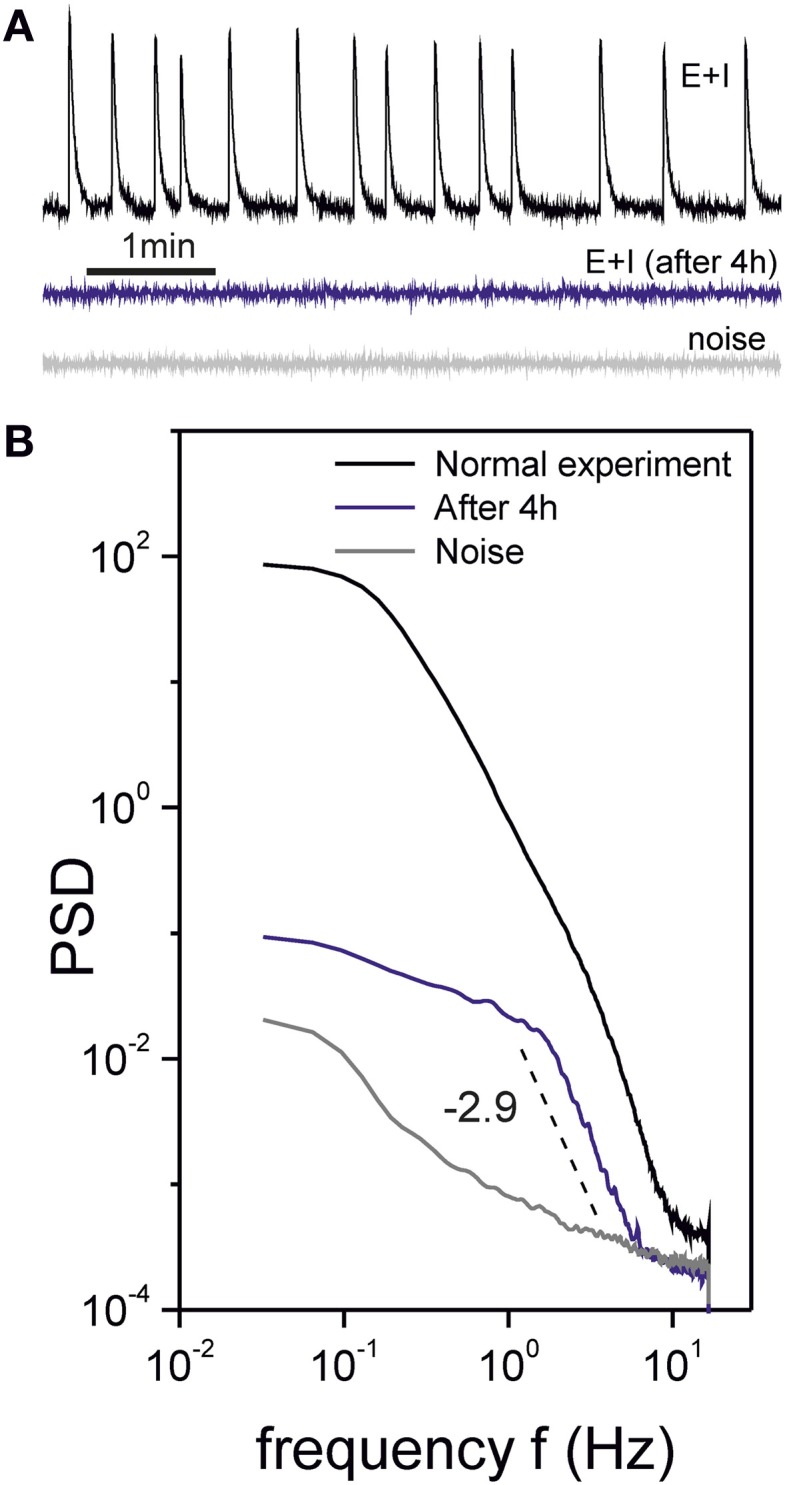
**Unhealthy cultures**. **(A)** Traces of fluorescence spontaneous activity in a “E+I” culture recoded in healthy (top) and unhealthy (center) conditions. The unhealthy state was induced by maintaining the culture under continuous light exposure for 4 h, which resulted in the death of several neurons. The unhealthy network showed individual neuronal firing but was deprived of bursting activity. The bottom trace shows the noise of the recording system. **(B)** Corresponding PSD curves. The unhealthy state shows a trend very different from both the healthy state and the noise, with a scaling at high frequencies that may arise from temporal correlations between individual neuronal firings.

Photo-damage in such an experiment induced neuronal death and severe disruption in the normal neuronal network behavior, which was evidenced by the extinction of bursting episodes. However, close inspection of the recordings showed that local activity, in the form of individual firing or groups of persistently active neurons, was still identifiable. Figure 10A shows traces of network-averaged fluorescence to compare the healthy and unhealthy states. We also show the fluorescence signal corresponding to the noise of the camera.

The corresponding PSDs of these measurements are shown in Figure 10B. Remarkably, the PSD for the deteriorated, unhealthy culture displays a neat scaling that is not masked by the bursts' structure. Also, the PSD is qualitatively similar in shape as the one for healthy cultures and [CNQX] = 800 nM. Interestingly, we measured clearly different exponents α. For the healthy and CNQX-drugged networks we consistently measured exponents of α ≃ 2.0, while for the unhealthy experiments we obtained α ≃ 3.0. Such a different values reveal different temporal correlations or dynamical modes in the network, and hints at the potential of PSD analysis to quantify the state of neuronal networks.

## 4. Discussion

Our experiments fall within the context of *functional multineuron calcium imaging* (fMCI), a technique based in the ability to examine network activity in large neuronal populations and with single-cell resolution (Stosiek et al., 2003; Ohki et al., 2005; Bonifazi et al., 2009; Takahashi et al., 2010a,b). fMCI has received substantial attention in the last years driven by the spectacular development of optogenetic tools and genetically encoded calcium indicators, which allow to monitor and probe neuronal circuits *in vivo* without the need of electrodes or other invasive measuring techniques (Yizhar et al., 2011a).

Given the challenge in fMCI to link the measured calcium fluorescence signal with the structural and dynamical traits of the underlying network, *in vitro* preparations have emerged as valuable platforms to probe neuronal circuitry and investigate the properties of the measured fluorescence signal. In this work we have utilized spontaneous activity in cortical cultures as the main measure to investigate the relation between activity, fluorescence signal and network connectivity. We have used two major approaches to access different neuronal circuitries, namely the monitoring of network development along 3 weeks and its controlled disintegration through application of CNQX. In both cases we observed distinct features in the shape of the fluorescence signal and its associated power spectrum density (PSD). The PSD could capture relevant events during development, revealed locality features in the neuronal network, and highlighted the presence of synchronous oscillatory modes within neuronal communities.

### 4.1. Fluorescence signal and power spectrum

The recorded fluorescence signal displayed different traits depending on both the age of the neuronal culture and its connectivity strength. First, young cultures under DIV ≲ 5 did not display bursts, and the networks dynamics was characterized by sparse individual neuronal firings of very low amplitude. We detected the presence of these events in the histograms of fluorescence amplitude (Figure 3B), which deviate from Gaussian distributions at high fluorescence values. However, the PSD curves corresponding to these “young” traces were similar to the ones obtained by measuring the noise of the camera. Hence, in very young cultures and with the experimental settings that we used in the present work, we could not use the power spectrum to quantify temporal correlations between neurons or other dynamical features.

Second, cultures at DIV ≳ 6 did show bursts, with a structure (amplitude, width and interburst timing) that depended on maturation. The corresponding PSDs reflected such variations, and we could detect GABA switch as well as the relative strength between excitation and inhibition by comparing the PSD curves of the “E+I” and “E-only” conditions (Figure 3). Also, the rise in bursting amplitudes during development was reflected in the PSD by a gradual increase in the average power (Figure 4). The PSD curves for mature cultures showed a rather good collapse with a slope α ≃ 2.5, indicating the advent of a more stable network state. Despite the variations from culture to culture, such a trend was systematic. Hence, in principle we could “guess” the developmental stage of a culture, and even some coarse properties, based in the average energy and slope of the PSD.

We must note, however, that the shape of the PSD arises from a complex combination of factors, including the fast jump in fluorescence at the beginning of bursting, the width of the bursts, the slow decay of fluorescence back to the resting state, as well as the time between burst. One would therefore need a detailed exploration of these different parameters to fully understand the information that the PSD can provide. Given the variety of bursting regimes that a neuronal culture can convey (Van Pelt et al., 2004b; Wagenaar et al., 2006a,b), such a exploration is a considerable endeavor.

As a third major remark, we observed distinct features in the PSD between the development of the network and its disintegration through CNQX. The former includes the growth and strengthening of connections, both locally and globally, and thus the overall network dynamics constantly evolve. The latter weakens homogeneously the excitatory connectivity in the network, leading to essentially a similar network dynamics with progressively reduced bursting. Hence, young cultures are not equivalent to fully disintegrated mature cultures. The two experimental approaches are therefore complementary and reveal distinct features. Indeed, a remarkable observation in the experiments with CNQX is that, for concentrations that led to almost no bursting at [CNQX] ≃ 800−2000 nM, we observed significant individual neuronal firing across the culture. Given the maturation of the network, these firings were of sufficient strength to exceed the noise of the system. Only in these conditions the PSD followed a scaling that we believe was capturing correlations between neurons (Figure 6).

The investigation of temporal correlations from PSD analyses is indeed a powerful concept since it may unveil dynamical traits of the network, e.g., in the form of synaptic inputs or intrinsic neuronal interactions (Thurner et al., 2003; Destexhe and Rudolph, 2004; El Boustani et al., 2009). The significance of the scaling by itself in our data, as well as the information that these correlations provide about the interplay activity-connectivity in the network, needs detailed investigation. Notably, the observation that healthy and unhealthy cultures exhibit different scaling exponents suggest that such studies could provide a basis to describe pathological or deteriorated cultures from the analysis of the PSD. In this context, an additional experimental tool that would provide valuable insight is the incorporation of connectivity guidance in the culture substrate, for instance in the form of biochemical fixation or physical trapping (Eckmann et al., 2007; Wheeler and Brewer, 2010). Dynamics in such “patterned cultures” substantially differ from standard ones due to the dictated connectivity (Shein Idelson et al., 2010; Tibau et al., 2013), and would possibly give rise to different temporal correlations.

### 4.2. Development and network traits

Several works in the literature have investigated the emergence of network-spanning bursting episodes during development. Consistently with our work, bursts were reported to appear by DIV 5–6 (Kamioka et al., 1996; Opitz et al., 2002; Wagenaar et al., 2006a), showing a low amplitude and irregular timing. These studies used micro-electrode arrays (MEAs) as activity-measuring technique, and also revealed that the activity contained both individual firing events and bursts. As said before, this individual spiking was also present in maturer networks (DIV ≃ 10 and older), and we actually used the valuable information that they provide to reconstruct neuronal connectivity in the context of Transfer Entropy (Stetter et al., 2012). Mature cultures exhibited stronger and more regular bursting as a consequence of the progressive maturation of synapses and the increase in their number (Muramoto et al., 1993; Kamioka et al., 1996; Opitz et al., 2002). Interestingly, we observed a stabilization in bursting amplitudes as well as a decrease in bursting firing frequency by DIV 18–20 (Figures 2D,E). These results are consistent with the studies of Van Pelt et al. (2004b,a) who reported that, in cortical cultures similar to ours, burst duration and firing amplitudes reached maximum values by DIV 18, to later stabilize or decrease as network evolved further.

The different spatial analysis of the PSD (Figures 7, 8) for mature cultures during network disintegration revealed strong inhomogeneities in the distribution of spectral energies, with compact spots of high energy. Spectral energy is directly linked to the amplitude of the bursts which, in turn, is related to the number of the elicited action potentials (Sasaki et al., 2008). If we assume that neurons firing with large bursting amplitudes have a higher input connectivity, then the combination of strong firing and spatial closeness identifies neuronal communities that are highly interconnected. The cohesion within a community is maintained up to complete disintegration of the network. Chiappalone et al. (2006) showed that spatially close neurons are progressively more functionally connected as the network matures; and Soriano et al. (2008) showed that, in CNQX disintegration experiments similar to ours, groups of neurons spatially close maintained their interconnectivity and collective firing when stimulated electrically.

Hence, we ascribe this spatial inhomogeneities in the PSD to the formation of highly conserved topological communities that maintain unique local features despite changes in global network dynamics. We indeed hypothesize that the communities observed by Chiappalone et al. (2006) are the same as our groups of “highly energetic neurons.”

### 4.3. High frequency synchronous oscillations

The PSD curves upon CNQX disintegration revealed the existence of high-frequency oscillations in the range 5–10 Hz, which were remarkably strong and spatially localized at particular concentrations of CNQX. These oscillations were observed solely in mature cultures and, in general, we detected them both in the “E+I” and “E-only” conditions. A detailed study of the fluorescence traces revealed that the oscillatory modes originated from activity within the bursts themselves. Interestingly, Shein Idelson et al. (2010) reported oscillations in small neuronal circuits formed by compact cell aggregates. They observed collective oscillatory modes within network bursts in the range 25–100 Hz, and the authors associated them to synchronous oscillations during the decaying phase of the network burst.

Our observed oscillations are markedly strong in localized communities, suggesting that the oscillations emerge as a result of recurrent activity within these communities. We found puzzling, however, the observation that the CNQX concentrations at which the oscillations had the highest amplitude coincided with sudden increases in global network dynamics. We suggest that the network may activate correction mechanisms at a critical connectivity weakening to prevent the deterioration of activity. These mechanisms may arise from local alterations in synaptic strength or connectivity, as well as from changes in the excitability of the neurons themselves.

It also may occur that these communities of oscillatory activity play a role in the network, for instance as centers for the initiation of activity. Orlandi et al. (2013) recently introduced the concept of “noise focusing”, the amplification and propagation of network background activity toward specific foci or basins of attraction where bursts ultimately initiate. It would be enlightening to investigate if there is a relation between these foci of burst initiation and our oscillatory communities.

Finally, we remark that these oscillations seem to be inexistent in young cultures (or too weak to be detected), which strengthens the argument that strong coupling within the cell community is required for their generation. An aspect that requires investigation, however, is what parameters tune the frequency of the oscillations, for instance by exploring the relative weight between AMPA, NMDA and GABA receptors. Shein Idelson et al. (2010) indeed showed that the oscillations disappeared altogether when GABA was fully blocked, which does not occur in our case.

### Conflict of interest statement

The authors declare that the research was conducted in the absence of any commercial or financial relationships that could be construed as a potential conflict of interest.
